# “As if we didn’t exist” – A participatory reflexive thematic analysis on next of kins’ experiences of their interactions with the psychiatric health care system in Germany

**DOI:** 10.1186/s12888-025-07481-0

**Published:** 2025-10-02

**Authors:** Nora Dietrich, Silvia Bahl, Sebastian Bayer, Susanne Kappesser, Thomas Klatt, Johanna Leona Kummetat, Sebastian von Peter, Sarah Schernau, Sven Speerforck, Laura Galbusera, Kolja Heumann

**Affiliations:** 1 Department of Psychiatry and Psychotherapy, Medical School of Brandenburg (MHB), Rüdersdorf bei Berlin, Germany; 2 Department of Psychiatry and Psychotherapy, Medical Faculty, University of Leipzig, Leipzig, Germany

**Keywords:** Next of kin participation, Next of kin perspectives, Informal caregiver, Psychiatric care, Participatory research, Reflexive thematic analysis

## Abstract

**Background:**

Involving next of kin in psychiatric care has been shown to significantly enhance the recovery process of the person in treatment as well as the wellbeing of the next of kin and aligns with clinical guidelines. However, despite broad recommendations, their participation remains inconsistent, and many next of kin report dissatisfaction with their involvement. Research predominantly focuses on psychiatric staff perspectives, leaving a gap in understanding how next of kin perceive their interactions with psychiatric professionals and institutions.

**Methods:**

This study is part of the participatory-collaborative research project “PazAng,” investigating the barriers and opportunities for systematic next-of-kin involvement in German psychiatric care. We conducted 15 guided semi-structured interviews with next of kin, and analyzed them using Reflexive Thematic Analysis. To ensure diverse perspectives, the participatory approach included both researchers with lived experience as next of kin and those without such experience.

**Results:**

We analyzed four major thematic fields shaping the experiences of next of kin in psychiatric care: “Feeling rebuffed vs. welcomed,” “Invisibility vs. feeling acknowledged,” “(Repeated) Powerlessness vs. Agency,” and “Paradoxical Assignment of Responsibility vs. Relief.” Some positive experiences were reported when staff actively included next of kin, provided triangulating communication, and facilitated collaborative discussions. However, such cases were the exception rather than the norm. The findings underscore the urgent need for a cultural shift towards greater inclusion and recognition of next of kin in psychiatric care.

**Conclusion:**

A systematic change in psychiatric care is required to facilitate meaningful next-of-kin participation. This includes clear institutional policies, more focus on relational aspects, and a redefinition of participation beyond caregiving responsibilities. A more proactive, inclusive approach to next of kin could enhance support for both individuals in treatment and their next of kin while improving overall psychiatric care outcomes.

**Supplementary Information:**

The online version contains supplementary material available at 10.1186/s12888-025-07481-0.

## Background

 A substantial and well-established body of research highlights the social-relational dimension of mental health that interacts with the individual one in complex ways [1]. For example, the social environment can act as a buffer against short-term and chronic stressor [1, 2], and positive social relationships can provide emotional security and enhance both quality of life and life satisfaction [3–5]. On the other hand, strained social relationships can contribute to the development of mental health crises such as depression [6, 7], while mental health crisis themselves can place considerable strain on the social environment [8]. It is therefore important to understand mental health and psychiatric treatment not only as individual or mere neurobiological experiences but also as socially embedded processes.

The involvement of next of kin in psychiatric care represents an essential component of a socially grounded approach to mental health and psychiatry. The term *next of kin* refers to individuals who have a close and trusting relationship with a person undergoing psychiatric treatment (see 2.2). Numerous studies demonstrate the effectiveness of involving next of kin in psychiatric care [9, 10]. Early involvement of next of kin has been shown to significantly contribute to recovery [11, 12] and has shown to improve the wellbeing of next of kin [13, 14]. It is therefore firmly embedded in many clinical guidelines [15–17] and aligns with the principles outlined in the UN Convention on the Rights of Persons with Disabilities [18]. As a result, the inclusion of next of kin is widely advocated in the research literature, both from ethical and economic perspectives [19].

Despite these broad recommendations, routine psychiatric care often fails to reflect this ideal [19]. Many next of kin report dissatisfaction with their experienced participation [19–22]. While several studies have explored barriers to next-of-kin involvement, they have predominantly focused on the perspective of psychiatric staff and the British health care system [19].In contrast, much less is known about how next of kin perceive their interactions with psychiatric staff and the psychiatric health care system especially in Germany.

This research gap is significant, as a deeper understanding of next of kin’s experiences may help to address their needs more effectively and to enhance their participation in psychiatric services. Moreover, it can be assumed that the relationship between next of kin and staff plays a crucial role in shaping the relationships between the person in treatment and psychiatric staff as well as the relationship between next of kin and the person in treatment [23]. Furthermore, there are legal differences regarding the role of next of kin within in psychiatric health care system between Great Britain and Germany. While the British Mental Health Act of 1983 integrates next of kin through the role of Nearest Relative [24], with some formal rights and structured involvement, in Germany, next of kin do not have institutionalized participatory roles in psychiatric care unless specifically appointed [25].

Therefore, this study investigates the following research question:

- How do next of kin perceive their interactions with psychiatric staff within the German health care system?

The aim is to gain insights into the experiences of next of kin and to derive recommendations for improving psychiatric care.

## Methods

### Design

This study is part of the participatory-collaborative research project “PazAng: Opportunities and barriers to the systematic participation of next of kin in the psychiatric treatment system in Germany.” This project is implemented jointly by the Brandenburg Medical School, Leipzig University Hospital, and the Berlin Association of Relatives and Friends of People with mental Crises (ApK – LV Berlin e.V.) and is funded by the German Research Foundation (DFG). To ensure that the entire research process is rooted in diverse and experience-based perspectives, researchers with different professional backgrounds, as well as researchers with and without personal experience as next of kin of people in psychiatric treatment, collaborated. Academic qualifications were not mandatory for the peer researchers; instead, extensive experience in self-advocacy and self-help, to make use of the collective forms of experiential knowledge. The participatory research framework also involved a steering board.

As part of the project, 30 guided interviews were conducted with next of kin [15], clinical staff [8], and persons in psychiatric treatment [7]. Since the interview material with next of kin was particularly rich, and to place a special focus on their perspective, the present manuscript exclusively reports the analysis of the 15 interviews with next of kin.

### Wording

The collaborative PazAng team and the participatory steering board critically examined the terminology commonly used in this field. We decided to use the term *next of kin* to refer to individuals who have a close and trusting relationship with a person undergoing psychiatric treatment. Drawing on Haraway’s (2016) concept of kinship, we understand kin not solely as biological relatives (*godkin*) but also as those we intentionally form bonds with (*oddkin*), emphasizing relationships beyond ancestry or genealogy [26]. While related studies often use the term *(informal) caregiver*, our discussions highlighted concerns about its implications. Some argue that this term diminishes the significance of the relationship by framing it as merely supportive and thus also constructs persons in treatment as dependents. Additionally, many next of kin do not identify with this label, as they perceive their caregiving responsibilities as embedded in their everyday lives rather than as a distinct role [27]. At the same time, alternative terms such as *relative* or *family* risk excluding friends or other trusted individuals.

We also decided to use the term *person in psychiatric treatment (PIT)* to describe individuals receiving psychiatric care. Alternative terms such as *service user*,* patient*,* person with a psychiatric diagnosis*,* person in mental health crisis* were considered. However, we chose *PIT* because it highlights the specific situation that defines the relationship between the three key stakeholders: psychiatric staff, the person in treatment, and next of kin. Additionally, this term avoids assumptions about the individual’s experience—for example, not everyone perceives themselves as being in crisis, identifies with a diagnosis, or considers themselves a user of a *service*. Thus, *person in psychiatric treatment* was considered less stigmatizing. Given that our research focuses on next of kin’s participation in the psychiatric healthcare system, this term provides an appropriate and inclusive descriptor. At the same time, it is important to acknowledge that many next of kin also play a crucial role for individuals who are not engaged in formal psychiatric treatment.

In general, we focus our study on adult persons in treatment and next of kin, considering the significant differences in history, settings, and requirements for child and adolescent psychiatry.

### Participants

The recruitment of interview participants was conducted through notice boards in clinics in Germany in Brandenburg and Leipzig (inpatient, day-care, psychiatric outpatient departments, and home treatment), direct contact by clinical staff, and distribution via self-help organizations.

Participants were selected based on the principle of maximum variation to ensure a diverse sample in terms of treatment settings (inpatient, day-care, outpatient, and home treatment), gender, age, and duration of involvement as next of kin. Furthermore, it was ensured that the participants were not recruited exclusively through self-help organization, following the assumption that this would reflect a specific sample. The criteria for the sampling strategy were thoroughly discussed within the participatory team. It was decided to select factors that emphasize the next of kin and their relationship to the person in treatment over medical data of the person in psychiatric treatment. Participants had to be at least 18 years old and have sufficient German language skills for the interview. The exclusion criterion was dementia in the affected individuals. This was based on the practical counseling experiences that next of kin of people with dementia often have a special role which is usually more connected to (physical) caregiving and less stigmatized.

The decision to conduct fifteen interviews was based on several factors: first, our commitment to in-depth, qualitative inquiry rather than representational generalizability; second, the principle of information power [28].

### Interviews

A semi-structured interview guide (see Additional file 1) was developed in several participatory workshops following the SPSS principle according to Helfferich (2011) [29]. It was designed to ensure that the interviews addressed specific thematic areas while allowing for flexible in-depth exploration. The thematic areas were *subjective (role) experiences with participation*,* structural and personal barriers*,* needs and requirements*,* meaning and benefits of participation* and were each introduced with an open introductory question and explored in greater depth as required.

The interviews were conducted by various team members of the collaborative PazAng team, interview quality was ensured by a workshop on interview techniques within the team. Interviews took place at locations chosen by the participants. Possible locations included self-help organization facilities, clinical settings, or participants’ homes. Participants received a compensation of 30 euros for their time.

After the interviews, participants completed a sociodemographic questionnaire, a pseudonymization code, a contact form, and provided their contact details for reimbursement. Additionally, the interviewers recorded their spontaneous impressions and any notable occurrences on a protocol sheet.

### Analysis

The interview data were coded and organized using MAXQDA (2022) [30]. Analytical procedures followed the principles of *Reflexive Thematic Analysis* (RTA) as developed by Braun and Clark [31], which conceptualizes theme development as a creative, interpretive, and reflexive process. This approach aligns with a *Big Q* qualitative paradigm [32] informed by social constructionist and critical realist assumptions, treating data not as objective reflections of reality but as contextually embedded and socially co-constructed narratives.

In accordance with the participatory character of the research project, the analysis began with a methodological workshop on Reflexive Thematic Analysis. Steps 1 and 2—data familiarization and initial coding—were carried out collaboratively by a multidisciplinary team with diverse disciplinary backgrounds (e.g. psychology, cultural studies, experiental knowledge through self-advocacy and self-help) and varying lived experiences as next of kin (e.g., sibling, partner, friend, or none). These team members (SB, SK, SBe, ND) met weekly in a research workshop format that enabled communicative validation and iterative reflection.

Theme development proceeded inductively and reflexively. The first generation of themes (Step 3) was conducted by ND, while the review and refinement of themes (Step 4) took place in multiple participatory workshops with broader team involvement (JK, KH, ND, SB, SK, SP, TK). Throughout, coding was understood not as a technical or purely descriptive task, but as an interpretive act in which researchers actively shaped and constructed meaning through engagement with the data.

The positionality of the research team was actively acknowledged and reflected upon during all stages of the analysis. In line with the principles of participatory research [33, 34], the diversity within the team was treated not as a bias to be corrected, but as an epistemological resource. Researchers brought different degrees of proximity to the subject matter—some identified as next of kin themselves, some researchers possessed cumulative experiential knowledge through their involvement in next of kin organizations, some worked as clinical staff. These differences were not neutralized, but embraced as enriching the interpretive process. Rather than seeking consensus or definitive codes, team discussions were used to deepen interpretive insights and to critically interrogate assumptions. This process reflects the reflexive ethos of both participatory and reflexive thematic methodologies.

Efforts were made to avoid tokenistic inclusion by fostering equitable participation. Power dynamics within the team were explicitly addressed and openly negotiated—for example, through shared authorship, collaborative analysis, and designated spaces for critical reflection. Experiential knowledge was seeked not to be treated instrumentally, but as equally valuable to academic expertise. Reflexivity, therefore, was not confined to a specific analytic phase but understood as an ongoing, layered process of institutional, personal, and epistemic self-interrogation.

For publication purposes, all interview excerpts were translated from German into English with careful attention to preserving meaning and context.

## Results

### Participants

Fifteen next of kin participated in the semi-structured interviews conducted between November 2023 and March 2025 in Germany. Five interviews were conducted by two peer researchers, seven interviews by two academic staff members with a psychotherapeutic background, and three by an academic staff member specializing in medical quality research.

We received many interview requests from next of kin within a short period, particularly from those who learned about the study through next of kin advocacy groups. The number of requests far exceeded the number of interviews we were able to conduct (Approximately twice as many), which may indicate the significance of the topic. We selected interview partners based on our predefined maximum variation sampling criteria, as far as this information was available beforehand.

Sociodemographic details of the interview participants are presented in Table 1. Two participants chose to take a break during the interview for emotional calming.

**Table 1:** Sociodemographic characteristics of N=15 next of kin

Characteristic	n	%
Age		
< 40 years	2	13.33
40-60 years	5	33.33
>60 years	5	33.33
Not indicated	3	20.00
Mean (SD)	56.33 (14.88)	
Gender		
Female	11	73.33
Male	4	26.67
Other	0	0.00
Kinship relation (multiple answers possible)		
Mother	5	33.33
Father	1	6.67
Sister	2	13.33
Son	1	6.67
Daughter	1	6.67
Partner, female	2	13.33
Partner, male	2	13.33
Friend	2	13.33
Aunt	1	6.67
Room mate	2	13.33
Duration of involvement as next of kin		
<5 years	2	13.33
>5 years	13	86.67
Mean (SD)	16.67 (9.82)	
Living in the same household		
Yes	2	13,33
Sometimes	2	13,33
No	11	73,33
Next of kin’s association (participation in activities or active engagement)		
Yes	10	66,66
No	5	33,33
Treatment setting (multiple answers possible)		
Inpatient Treatment	15	100
Day clinic	5	33.33
Home Treatment	3	20
Outpatient Treatment	10	66.66
Experiences of coercive treatment	5	33.33

### Reflexive thematic analysis

From the interview material, we analyzed four key thematic fields that shape the experiences of next of kin in and their interactions with the psychiatric care system: “Feeling rebuffed vs welcomed”, “Invisibility vs feeling acknowledged”, “(Repeated) Powerlessness vs Agency” and “Paradoxical Assignment of Responsibility vs Relief”.

When generating and reviewing the themes (Step 3 and 4 of the Reflexive Thematic Analysis) we engaged in extensive discussions. In response to the question of what relatives experienced in their interactions with the psychiatric care system, we predominantly (though not exclusively) received accounts of negative experiences. This aligns with previous studies [19–21] as well as the initial preliminary findings of our subsequent quantitative study. At first, we considered presenting only these negative experiences, as they were so prominent. At the same time, we recognized that the positive experiences offered important insights into potential areas for change. For this reason, we decided to capture a broad range of experiences within the thematic fields. This carries the risk that the experiences might appear “too balanced.” Therefore, we want to emphasize that negatively perceived interactions were particularly prominent in the interviews. Nevertheless, we believe that the positive experiences contain valuable opportunities for change from which we can learn. For this reason, we chose to analyze thematic fields.

#### Feeling rebuffed vs. welcomed

In many accounts from the interviewed next of kin, narratives were present that described contacting clinical staff as a challenge. Next of kin expressed feeling the need to “push” (INTERVIEWEE 4), “fight” (INTERVIEWEE 11), “insist” (INTERVIEWEE 4), or “make an enormous effort” (INTERVIEWEE 11), only to engage in conversations with clinical staff. As a consequence, they had the impression of being perceived as “annoying” (INTERVIEWEE 11) while otherwise not being heard. Some next of kin described feeling “brushed off” (INTERVIEWEE 4) or rebuffed:

*“And yes*,* the last few times*,* yes*,* that didn’t happen at all anymore (…) And I have the feeling that even when you ask for a conversation*,* you don’t get a call back. (…) And I felt very excluded.”* (INTERVIEWEE 11).

*“I wrote emails*,* I called*,* I asked*,* I did everything. There was nothing*,* no response. None. (…) It didn’t work. (…) I: How did that make you feel? B: Sad and angry.”* (INTERVIEWEE 8).

Confidentiality was frequently discussed in this context and cited in many interviews as a reason for the difficulties in involvement. On the one hand, some next of kin perceived it as an understandable and legitimate obstacle to contact, as clinicians had to “follow the guidelines” (INTERVIEWEE 6) and aim to “protect the patient’s trust” (INTERVIEWEE 12). At the same time, relatives also describe the impression that some clinicians hide behind confidentiality and use it as an excuse for exclusion of next of kin:

*" I would always say: This whole ‘I can’t talk to you’ really just means ‘I don’t want to talk to you.‘”* (INTERVIEWEE 14).

*“Data protection and all that—things that are actually good are being used against you.”* (INTERVIEWEE 6).

Many interview participants reported that they, as next of kin, had to be the primary ones to initiate contact:

*" in all these 20 years? I honestly can’t remember a single time when they reached out. The contact always came from me. Always.”* (INTERVIEWEE 7).

For this reason, various next of kin expressed the desire for clinical staff to actively reach out to them.

Although less frequently, some interviews also contained accounts of a welcoming approach. Next of kin expressed satisfaction when staff were easily accessible and conveyed a sense of appreciation and being welcome through friendly communication. One example related to the possibility to call at any time for follow-up questions, which was perceived as relieving:

*“They were always like: ‘call anytime’. I just had to send an email or something*,* and they would say: ‘Would it work for you if I call you today at 3:00 PM? You can also come by (…)’ Always.”* (INTERVIEWEE 14).

*“And I experienced the institution itself as very friendly. I could only speak to them on the phone. At first*,* I accidentally called the wrong department*,* but they transferred me. And it was all very calm*,* very composed*,* and also friendly.”* (INTERVIEWEE 6).

#### Invisibility vs. feeling acknowledged

Another theme that we analyzed from our interviews focuses on the experiences of next of kin feeling invisible, barely acknowledged, and passively ignored in their significance and own needs:

*“Because*,* I mean*,* we as relatives are the ones who catch and support the affected individuals afterward. And I feel that this capacity or potential is not sufficiently recognized by the clinics.”* (INTERVIEWEE 11).

*“Well*,* as a relative*,* (…) you’re not really acknowledged by the doctors.”* (INTERVIEWEE 7).

*“In—I’ll put it bluntly—15 to 20 years of living with this illness*,* those were rare exceptions. Otherwise*,* no one ever really wanted to know anything from me.”* (INTERVIEWEE 9).

*“I feel like we’re always the last priority.”* (INTERVIEWEE 5).

One interviewee connected this frequent neglect of next of kin with patriarchal power structures and the devaluation of care work:

*“This is just the typical care work done by women—it’s never worth anything*,* is it?”* (INTERVIEWEE 14).

The experience of feeling invisible was present in various relationship roles. However, it appeared to be especially prominent for certain groups of next of kin, especially siblings and (adult) children:

*“The thing with siblings—we somehow always get forgotten*,* as if we didn’t exist.”* (INTERVIEWEE 13).

*“And I always wonder if they even think about the fact that a family is more than just parents. (…) I’m standing on the outside here*,* not knowing whether they ever think about the fact that we (siblings) exist too?”* (INTERVIEWEE 13).

*“Yes*,* but I—I don’t think anyone spoke with us children. (…) I think I was just so shocked. (…) I wish someone had talked to me and at least told me what they thought had happened or something like that*.” (INTERVIEWEE 2).

Next of kin also pointed out that *“friends or partners somehow always seem to be overlooked”* (INTERVIEWEE 13) and emphasized that *“children should also have someone to turn to.”* (INTERVIEWEE 12).

In contrast, some interviewed next of kin described situations, in which clinical staff addressed their needs and valued their perspectives:

*“I felt like the doctor really listened carefully to how I was feeling about the situation—about how deeply worried I was. (…) I really felt like*,* okay*,* this is being taken seriously. And in the end*,* I actually (laughs) got help.”* (INTERVIEWEE 3).

*“There were often conversations between the three of us (clinician*,* next of kin*,* person in treatment) about how to handle things—where my perspective mattered just as much as his (person in treatment)*,* and we worked out solutions together.”* (INTERVIEWEE 8).

*“(Name of clinician) said: ‘It really seemed like you were starving for the chance to talk about this and to get some input.’ And that’s exactly how it was*,* right? It all just had to come out.”* (INTERVIEWEE 10).

#### (Repeated) powerlessness vs. agency

Many accounts from the interviewed next of kin revealed strong feelings of powerlessness and external control in their interactions with the psychiatric care system. This theme is particularly rich in metaphors in the interviews:

*“I just saw myself in checkmate. No one has a duty to provide information*,* no one has any obligation to me at all.”* (INTERVIEWEE 9).

*“I felt like I was in a vacuum*,* with zero options for action.”* (INTERVIEWEE 9).

Powerlessness was also shaped by the experience that it was the person in treatment and/or the clinical staff who decided on the inclusion of next of kin, while next of kin themselves felt to have little decision-making power in this regard:

*“And I just feel abandoned because I don’t know the diagnosis or anything at all. I tried to contact the doctor. The doctor cited confidentiality. My husband*,* in the meantime*,* said he didn’t want the doctor to give me any information.”* (INTERVIEWEE 12).

*“So*,* I experienced it as powerlessness because (…) the person in treatment is so protected—which*,* of course*,* is good. But at some point*,* I asked myself*,* if the person in treatment is protected in this way by their right to self-determination and human rights*,* to put it bluntly*,* where is the protection for me?”* (INTERVIEWEE 9).

*“And*,* of course*,* what the patient wants is what matters most.”* (INTERVIEWEE 15).

*“And at that moment*,* well*,* that’s what (name of person in treatment) told me*,* they didn’t really feel an urgent need for it (joint conversations). And so*,* it just didn’t happen. Not until the end. I asked about it multiple times*,* and it just didn’t happen.”* (INTERVIEWEE 1).

Some next of kin also described that the decisions of the person in treatment changed unpredictably, thereby restricting their ability to communicate with clinical staff:

*“And sometimes the doctors were allowed to give information*,* and then suddenly they weren’t. Depending on her mood*,* she (person in treatment) said*,* ‘Yes*,* go ahead*,*’ or ‘No*,* you can’t.’ And I believe that in those moments*,* she simply lost control. She was trying to exercise power.”* (INTERVIEWEE 9).

Many next of kin not only described a lack of agency in their attempts to establish contact but also in their direct interactions with clinical staff. The language used conveyed an impression of a power imbalance to which next of kin had to submit. For example, one person described their brief interaction with a doctor as a *“one-time hearing”* (INTERVIEWEE 9) for an external anamnesis:

*“As in: ‘What we hear and observe here does not match what we diagnose.’”* (INTERVIEWEE 9).

Descriptions of clinical staff, especially doctors, reinforced this impression:

*“They come in*,* boom*,* boom*,* boom*,* boom*,* boom*,* and then they’re gone. And you wait two hours*,* and then you might get five minutes*,* if that.”* (INTERVIEWEE 3).

*“He came over with a sour expression—just like these doctors always rush through the wards*,* avoiding eye contact with everyone*,* especially next of kin.”* (INTERVIEWEE 14).

One interviewee also noted the hierarchy in care work, pointing out that unlike clinical staff, next of kin are not compensated for their caregiving efforts:

*“Support meetings (…) I’m the only one who doesn’t get paid for them*,* I mean*,* one must be say. The professionals all get paid for it.”* (INTERVIEWEE 5).

Next of kin described feelings of powerlessness not only in their interactions with the psychiatric care system but also at the onset of the illness itself:

*“You’re stuck between a rock and a hard place and have no idea which way is up or down or how to handle the situation.”* (INTERVIEWEE 12).

*“It was just this huge*,* empty bubble where I had no clue what to do next.”* (INTERVIEWEE 13).

Within the psychiatric care system, many next of kin felt abandoned with these emotions of helplessness. In this way, the sense of powerlessness appears to be reinforced and solidified by the experience of isolation within the psychiatric context. For this reason, we decided to conceptualize the theme as ‘repeated powerlessness’.

In contrast, some next of kin described experiences in which the contact with the psychiatric care system helped them to regain a sense of agency. One interviewed mother who had experiences within home treatment services described how interactions with clinical staff allowed her to learn about herself and the illness. This experience helped her *“move out of the passive role”*.

*“As a next of kin*,* you’re not just sitting like a rabbit in front of a snake*,* waiting for the next catastrophe. Instead*,* you are consciously included*,* informed*,* and some of your fear is taken away.”* (INTERVIEWEE 10).

Some other next of kin reported positive experiences in which clinical staff facilitated three-way conversations (between next of kin, persons in treatment, and clinical staff), helping to counteract feelings of isolation and helplessness. These conversations made it possible to *“talk about things we otherwise wouldn’t discuss.”* (INTERVIEWEE 10)

*“And I always found the clinic very helpful*,* sort of like a mediator*,* bringing us together and helping us make agreements.”* (INTERVIEWEE 11).

*“Otherwise*,* it’s really difficult to have a dialogue. And I always felt that these joint conversations with the doctor and the social worker were incredibly helpful. My daughter—she was only able to concentrate on them for a limited time. But during those moments*,* she did focus. And I found that incredibly helpful because outside of those discussions*,* she was back in her own world. And I couldn’t reach her there.”* (INTERVIEWEE 11).

#### Paradoxical assignment of responsibility vs. relief

Some next of kin described experiencing a frustrating double heteronomy: during treatment, they were expected to stay out of the process, but afterward, caregiving responsibilities fell back on them—partly due to inadequate outpatient support structures:

*“I always experience it like this: my daughter is in the clinic—that much I still know. And then I am largely cut off from information. But when she is discharged*,* I am often the one who has to make sure that she has somewhere to live*,* that she can organize her livelihood*,* and that things continue in some way outside of the clinic.”* (INTERVIEWEE 11).

*“In the end*,* the next of kin are the only ones left to react somehow.”* (INTERVIEWEE 7).

*“You are completely left alone. And then nothing happens at all. If I didn’t have the financial means to pay 100 euros per session for therapy for four to eight months*,* then nothing would happen. My son would just sit there for four to eight months without therapy.”* (INTERVIEWEE 10).

*“We were not included*,* but still*,* we had to do all the work.”* (INTERVIEWEE 2).

The expectation that next of kin should take on a significant share of caregiving responsibilities after increasingly shorter hospital stays seems paradoxical—especially considering that they sometimes feel scrutinized and pathologized by clinical staff during treatment:

*“When you talk to the doctors at the beginning*,* you also get diagnosed yourself.”* (INTERVIEWEE 14).

*“And also*,* the doctors*,* when they don’t know what to do*,* they quickly look for blame elsewhere*,* right? So*,* for example*,* with the next of kin*,* who supposedly always do everything wrong*,* who then build these toxic relationships*,* or whatever*,* right?”* (INTERVIEWEE 5).

In contrast, few treatment experiences stand out in which mental illnesses and the role of next of kin were destigmatized—something that next of kin perceived as relieving:

*“It was about explaining: (…) I understood it as: ‘Don’t drive yourself crazy. Your son doesn’t have anything lifelong—he just needs proper treatment. (…)’ That really took a lot of weight off my shoulders.”* (INTERVIEWEE 10).

*“And it was a completely new perspective on the terrifying term ‘psychosis.’ I started reading widely on the topic*,* on the recommendation of (clinician’s name)*,* and what I read really resonated with me. Many things I had previously suspected—like the idea that it’s likely a network or systemic issue—were confirmed through the theories*,* experiences*,* and reports I read.”* (INTERVIEWEE 10).

This interviewee also described how it was helpful that a peer support worker made her aware of her own need for support:

*“Several people*,* especially the peer supporter*,* whom I found very helpful*,* kept telling me: ‘You need to seek help for yourself. Even if you seem incredibly strong*,* organizing everything and working and so on—you need to take care of yourself. You also need support.’ Someone has to actually say that to you*,* because you always think you’re completely invulnerable.”* (INTERVIEWEE 10).

Next of kin also valued opportunities for exchange with other next of kin—through partly facilitated support groups in clinics:

*“That was very relieving. And also realizing how other parents are doing. It was really good for me to be able to go there. (…) Simply realizing that I am allowed to set boundaries*,* you know?”* (INTERVIEWEE 4).

## Discussion

The results of our Reflexive Thematic Analysis illustrate how next of kin experience interactions with the psychiatric health care system. These experiences range from significant communication barriers to occasional instances of appreciative and empowering interactions. These differences raise critical questions about the practice of involving next of kin in psychiatric institutions and highlight potential avenues for improved ways of collaboration.

The frequently reported difficulties of next of kin in engaging with clinical staff primarily point at a lack of a suitable care context for effective participation. Thus, the described feelings of having to “push”, “fight”, and “insist” reflect a fundamental experienced asymmetry in interactions: next of kin often find themselves in a supplicant position, while both clinical staff and the person of treatment may act as gatekeepers. This suggests that many next of kin at least feel that clinical staff does not wish to engage with them, aligning with the findings of other studies that next of kin often struggle to establish contact with psychiatric staff and frequently feel to be seen as “troublemakers” [35]. Another study similarly reports that next of kin must fight for attention and, as a direct consequence, are often perceived as demanding and intrusive [36].

This images and stereotypes have longstanding historical roots: Scherer and Lampert (2017) describe how, until the 1950 s, the involvement of next of kin was seen rather as interfering with “medical affairs,” reflecting a psychiatric care system that operated largely isolated from the everyday life [37]. While psychiatry has undergone significant changes since then, a historically ingrained mistrust toward next of kin seems to persist. In the context of eating disorders, for instance, Herpertz-Dahlmann and colleagues critically refer to a long-standing practice of “parentectomy”— the deliberate exclusion of parents from psychiatric treatment. Although this approach is no longer in line with current guidelines, it may have contributed to a historically rooted reluctance to involve next of kin in psychiatric care [38].

This situation is also well exemplified by the recurring discussion of confidentiality in our interviews. Some next of kin reported feeling that confidentiality regulations were used by clinical staff as a pretext to avoid their active participation. Other studies have referred to this as a “confidentiality smokescreen” [35] and described the resulting tensions between next of kin, the person in treatment, and clinical staff [39]. Clearly,, the autonomy rights of the person in treatment must be upheld in clinical settings — especially given that their individual rights have, without doubt, been historically violated. This is in line with many interviewees—including many who voiced concerns about this practice of exclusion—stressing a fundamental understanding of the importance of confidentiality. The desire of next of kin to be involved should therefore not be mistaken for a disregard of the rights and interests of the person in treatment. Rather, a clear wish emerged that clinical staff should actively (and repeatedly) ask the person in treatment about their preferences and ensure that the participation of next of kin is consistently acknowledged and encouraged in a nuanced manner—just as clinical staff does when addressing disagreements, for example, regarding medication. If, despite these efforts, no contact is desired, clinical staff can still provide support to next of kin in ways that comply with data protection regulations (e.g., UK best practice: Slade et al. (2007)).

Another key finding is the repeatedly reported feeling of invisibility and isolation. Next of kin often feel that their significance is not acknowledged and that their perspectives and expertise are treated as secondary. This aligns with previous research [40–42]. Abou Seif and colleagues describes next of kin even as “invisible experts,” who are expected to cope with the situation on their own. Particularly siblings, children, or close friends of people in treatment frequently feel unaddressed, suggesting that existing concepts of participation primarily focus on parents [43].

One key reason for the continued neglect of the importance of next of kin is the ongoing dominance of medically oriented models of mental disorder [27]. These models primarily conceptualize psychosocial crises as individual (often neurobiologically caused) problems rooted within the affected person in treatment [44]. As a result, systemic and interpersonal dynamics, as well as their broader context, are often overlooked. This has significant consequences for the involvement of next of kin in psychiatric care, as they are frequently not recognized as relevant actors.

The reported invisibility of next of kin in psychiatric contexts also points to a gendered dimension. It is often women who take on the role of next of kin [21] and assume caregiving responsibilities. This pattern reflects a broader societal dynamic in which care work is both gendered and devalued [45, 46]. From a feminist and critical-analytical perspective, this suggests that the marginalization of next of kin is not solely the result of institutional structures within psychiatry, but is also intertwined with patriarchal gender norms that position women as primarily responsible for care—while simultaneously denying them authority and legitimacy within clinical settings [45]. Mothers in particular bear a double burden: they not only carry out the majority of emotional care work, but also frequently face systemic suspicion—being held partly responsible for the development of mental illness, while at the same time being excluded from meaningful participation in treatment processes [47].

Our findings suggest that many next of kin desire a more active role from clinical staff in initiating contact. The reported experience that next of kin usually had to initiate contact reinforces the sense of unequal responsibility. A systematic, proactive outreach by clinical staff could bridge this gap and lower the threshold for active involvement. Such an approach aligns with studies demonstrating the attitudes and overall culture of an institution to be the decisive factors in making next of kin feel welcome [19]. Thus, rather than requiring specific interventions, a cultural shift toward a more inclusive environment for next of kin in psychiatric institutions may be necessary first. In this context, it is important to acknowledge that next of kin not only play a crucial role in supporting a person in treatment but also have own needs that should be addressed. Dörner and colleagues propose a radical shift in this respect: clinicians should not only feel responsible for the person in treatment but for the entirety of a “social field” considering not collaborating with next of kin a “medical malpractice” [48].

Furthermore, our interview data demonstrates that next of kin often experience a profound sense of powerlessness in their interactions with psychiatric institutions. This finding is also in line with previous studies [36, 49, 50]. The reported exclusion appears to exacerbate the helplessness that many next of kind already felt at the onset of the illness. Particularly problematic is the recurring experience that next of kin are expected to take on full caregiving responsibilities after the discharge of people in treatment, after having been barely involved during this treatment. This structural inconsistency points to a paradoxical assignment of responsibility: next of kin are expected to step back during treatment but assumed to take-over a crucial role post-discharge [51, 52].

Critical voices argue that while the deinstitutionalization of psychiatry is a desirable development, its implementation—possibly in conjunction with the privatization of the health care sector—has shifted caregiving responsibilities increasingly onto next of kin without providing them with adequate means or support to step in [52]. Lloyed and colleagues highlight that discharge from psychiatric care does not necessarily imply patient recovery; rather, it often results in a transfer of tasks to next of kin [53]. This situation creates a paradoxical dual role for next of kin: on the one hand, as stated, they are excluded from the treatment process and may even be (partially) blamed for the illness of the person in treatment, while on the other, they are expected to take-over responsibility for caregiving once this person is discharged [51].

Valuable insights could also be drawn in our study from the positive experiences of interaction of the next of kind interviewed, even though these were far less prominent. In particular, when clinical staff proactively reached out, created an appreciative communication climate, or ensured low-threshold accessibility, this was perceived as relieving and supportive. Next of kin reported that their communication with clinical staff had strengthened their sense of agency, particularly when they had received information that enabled them to develop an understanding of the crisis. Triangulated forms of conversations and exchange between the person in treatment, next of kin, and clinical staff facilitated exchanges that had not previously been possible between the person in treatment and their next of kin. Moreover, de-stigmatizing perspectives, the proactive identification of support needs, and the opportunity for peer-level exchange were perceived to be significant in alleviating burden.

These positive examples, also consistent with previous studies [49]demonstrate that clinical practice can offer helpful approaches that enable constructive involvement of next of kin. Such approaches can positively impact their sense of agency, overall well-being, and improve the relationships between the person in treatment, their next of kin and clinical staff.

One promising avenue could be the systematic implementation of triangulated exchange formats that regularly involve next of kin, establish pathways for interactions, and address individual support needs. Interview findings also emphasize the structural dimension of these possibilities: next of kin involved in home treatment care systems with a focus on supporting the network reported particularly positive experiences in our study. This may suggest that approaches that takes place within the living environment may facilitate the involvement of next of kin. The Open Dialogue approach developed in Finland might represent a valuable concept in this context [54]. Additionally, next of kin in the interviews repeatedly expressed a desire for more proactive outreach services and long-term support for the person in treatment, emphasizing the importance of maintaining continuity in relationships with clinical staff. However, a fragmented care landscape is seen as a hindering factor for participation [51], as next of kin must repeatedly adapt to new points of contact and face the absence of reliable structures for their involvement.

### Strengths and limitations

The present study is characterized by a participatory approach and an interdisciplinary integration of findings, allowing for the inclusion of different perspectives and the generation of practical insights. The diversity in demographics and experiences among participants enabled the identification of common experiences across different contexts, particularly regarding varying kinship roles. However, there are some limitations: the recruitment also through next of kin organizations is likely to result in the participation of particularly engaged individuals. This may also be a strength simultaneously: the participants may already be particularly reflective and analytically sharpened as a result of their involvement, which may also be reflected in the rich interview material. In our interviews, satisfaction with participation as a next of kin did not appear to be related to whether participants were recruited via next of kin organizations or not. Our sample is characterized by an especially high representation of mothers being in line with previous studies [21]. All interviewees had experience with inpatient care as next of kin; most also had experience with other forms of treatment, particularly outpatient care, but also day clinics and home treatment. As a result, the findings of this study do not allow for clear conclusions about setting-specific differences in experiences. Further research is needed to systematically compare the experiences of relatives across different treatment settings. Additionally, people with a migration background were underrepresented. Nevertheless, a broad recruitment strategy, including outreach through clinical staff to visitors of hospitalized people, ensured a certain level of diversity also beyond the mere recruitment of participants of next-of-kin organizations. Despite these efforts, and given the self-selection recruitment strategy, it remains possible that individuals with strong opinions or negative experiences were more likely to participate in this interview study.

### Practical implications

Our findings indicate that a cultural shift in psychiatric care services is necessary to foster more effective and meaningful forms of next of kin involvement. Establishing a welcoming culture—beginning with basic, friendly interactions— can significantly improve their engagement. As Dörner and colleagues suggests, clinical staff should not only feel responsible for the person in treatment but for the entire relational field surrounding them [51]. Theoretical frameworks must integrate next of kin without placing blame, emphasizing a social approach rather than a guilt-driven discourse. Participation concepts should address the relational needs of next of kin, rather than viewing them merely as unpaid healthcare resources. This requires a more structurally based recognition and development of multi-person settings, including accounting systems that acknowledge and renumerate next of kin participation and the systematic training in this respect for clinical staff.

Beyond these conceptual and infrastructural barriers, psychiatric professionals also face rather practical challenges in involving next of kin into care processes. While many clinicians support family next of kin involvement in principle, they often feel uncertain about how to implement it effectively [55]. Studies indicate a lack of clear guidance defining the scope and priority of next of kin participation, leading to their frequent marginalization [55]. Additionally, heavy workloads, staff shortages, and financial pressures result in next of kin work being perceived as an additional, non-essential task [19]. Without addressing these structural constraints, next of kin involvement risks remaining a rhetorical commitment rather than a practiced reality.

Last but not least, clearer guidance is needed regarding confidentiality and the various roles of next of kin during involvement. A proactive approach—where people in treatment are actively encouraged to consider information-sharing and next of kin are informed about confidentiality regulations—could help mitigate uncertainties. Addressing the concerns and anxieties of both next of kin and the person in treatment is essential to fostering a more inclusive and supportive psychiatric care environment.

## Supplementary Information

Supplementary Material 1.

## Data Availability

The datasets used and/or analysed during the current study are available from the corresponding author on reasonable request in the original language (German).
