# Supplementary material for: “As if we didn’t exist” – A participatory reflexive thematic analysis on next of kins’ experiences of their interactions with the psychiatric health care system in Germany
Source: BMC Psychiatry. 2025 Oct 2;25:919. doi: 10.1186/s12888-025-07481-0 (PMC12490076; doi:10.1186/s12888-025-07481-0)
Supplement: Supplementary file 1 — Supplementary Material 1. [file 12888_2025_7481_MOESM1_ESM.pdf]

Leitfrage	Mögliche Nachfragen zur Unterstützung	Aufrechterhaltungs- und Steuerungsfragen
**Einstieg**		
(s.o.) Haben Sie vorab noch Fragen? Schön, dass Sie da sind. Wie ist es dazu gekommen, dass Sie heute an dem Interview teilnehmen?		
**Subjektive (Rollen-) Erfahrungen und Praxis des Einbezugs**		- Möchten Sie dazu noch etwas mehr erzählen?<br>- Haben Sie ein Beispiel für mich?<br>- Und dann? Wie ging es dann weiter?<br>- Wie war das mit….?<br>- Wie war das für Sie?
Was haben Sie im Kontakt zum psychiatrischen Versorgungssystem erlebt als Angehörige? Gibt es eine konkrete Erfahrung/ Situation, über die Sie berichten möchten?	- Worüber wurde gesprochen?<br>- Worüber nicht?<br>- Wie ging es Ihnen damit?<br>- Was denken Sie, was die Kliniker:innen von Ihnen als Angehörige erwartet haben?<br>- Welche Rolle kam Ihnen dabei zu?<br>- Wie hat sich während der Behandlung die Beziehung zu Ihrem Angehörigen verändert?<br>- Wenn passend: Welche Rolle hätten Sie spielen können?	
**Strukturelle und persönliche Barrieren und Tabus**		
Inwiefern haben Sie Schwierigkeiten dabei erlebt, in die Behandlung einbezogen zu werden?	- Welche Schwierigkeiten waren das?<br>- Falls passend: Was meinen Sie, warum haben Behandler:innen Sie so wenig einbezogen?<br>- Inwiefern haben Sie Vorbehalte und Befürchtungen bei Ihrem Angehörigen erlebt?<br>- Haben Sie selbst Befürchtungen gehabt, einbezogen zu werden?/ Was sind Ihre Befürchtungen bzgl mehr einbezogen werden?<br>- Welche Rolle spielen zeitliche und finanzielle Gründe?	
**Bedürfnisse und Bedarfe bzgl Einbezug von Angehörigen**		
Auf welche Art und Weise möchten Sie in die Behandlung einbezogen werden?	- Was wäre für Sie gut gewesen?<br>- Was hätten Sie gebraucht?<br>- Was braucht es dafür? (Klinikstrukturen, finanzielle Strukturen, persönliche Ressourcen)<br>- Was sollte dabei nicht passieren?<br>- Was denken Sie, was sich Ihr Angehöriger wünscht (bzgl des Einbezugs)?<br>- Haben sich Ihre Wünsche an die Behandler:innen über die Zeit verändert?	
**Abstrakt: Sinn und Nutzen des Einbezugs von Angehörigen in die Behandlungssituation**		
In welchen Situationen ist aus Ihrer Sicht die Beteiligung von Angehörigen während der Behandlung wichtig?	- Warum? Wozu?<br>- In Welchen Situationen nicht?	
**Abschluss**		
Jetzt würde mich noch interessieren, ob es Dinge gibt, die Ihnen wichtig sind, über die wir noch gar nicht gesprochen haben?		
